# Insensitivity as an Adaptive Psychological Resource for Mental Health: Development and Validation of a Scale Among Chinese University Students

**DOI:** 10.3390/bs16091612

**Published:** 2026-09-09

**Authors:** Zeyu Lei, Bo Liu, Tonglin Jin, Yuntena Wu

**Affiliations:** School of Psychology, Inner Mongolia Normal University, Hohhot 010022, China; leizeyu_psy@163.com (Z.L.); liubopsy@163.com (B.L.); psyjin@sina.com (T.J.)

**Keywords:** adaptive insensitivity, college students, reliability, validity, rumination, depressive symptoms

## Abstract

Depression and other mental health problems are increasingly prevalent among university students, highlighting the need to identify psychological resources that support mental well-being and early prevention. Adaptive Insensitivity has recently been proposed as a potentially adaptive psychological resource; however, no validated instrument is currently available to assess this construct. This study developed and validated the Insensitivity Scale (IS) among Chinese university students and further examined the mediating role of rumination in the relationship between insensitivity and depressive symptoms. An initial item pool was generated through word association tasks, open-ended surveys, online text analysis, and semi-structured interviews, and the resulting dimensions were subsequently organized and interpreted within the ABC model. The psychometric properties of the IS were evaluated across multiple independent samples using exploratory and confirmatory factor analyses, reliability analyses, and validity assessments. The final 15-item IS comprised three dimensions, Emotional Cognitive Management, Interpersonal Boundary Management, and Behavioral Response Management, and demonstrated satisfactory structural validity, internal consistency, test–retest reliability, and criterion-related validity. Insensitivity was positively associated with psychological resilience and regulatory emotional self-efficacy and negatively associated with emotional vulnerability, anxiety, and depressive symptoms. Rumination partially mediated the relationship between insensitivity and depressive symptoms, accounting for 52% of the total effect. These findings provide preliminary evidence that insensitivity is associated with better mental health among university students and may represent a potentially complementary perspective on adaptive psychological functioning. The IS demonstrated satisfactory psychometric properties for assessing individual differences in insensitivity and may facilitate further research on its role in psychological adaptation and mental health among university students.

## 1. Introduction

Mental health has become a global public health priority and a central component of the 2030 Sustainable Development Agenda, particularly Sustainable Development Goal 3 (SDG 3), which seeks to ensure healthy lives and promote well-being for people of all ages. Meanwhile, rapid advances in intelligent technologies have profoundly reshaped modern society, accelerating the pace of daily life while creating unprecedented opportunities as well as new psychological challenges (Ye & Cui, 2023). As individuals become increasingly exposed to chronic stress, information overload, and frequent social evaluation, maintaining psychological well-being has become progressively more difficult, contributing to rising levels of emotional dysregulation and mental health problems (Tsujimoto et al., 2024). Consistent with this global agenda, China’s 15th Five-Year Plan for National Economic and Social Development identifies mental health promotion as a national priority by emphasizing mental health education, strengthening mental health and psychiatric services, and advancing the early identification and comprehensive intervention of common mental disorders and psychological problems among key populations (Xinhua News Agency, 2026). Accordingly, considerable research has focused on strengthening psychological resilience (Qiao & Ye, 2026), enhancing stress resistance (He et al., 2025), and improving emotion regulation strategies (Wang et al., 2023) to help individuals cope with adversity and recover from psychological distress. Although these approaches have demonstrated substantial benefits, they primarily target the regulation or remediation of emotional difficulties after they have emerged. Comparatively less attention has been devoted to psychological resources that may reduce the psychological impact of excessive environmental stimulation, attenuate the initial intensity of emotional reactions, and prevent emotional exhaustion before psychological distress develops. Identifying such preventive psychological resources may therefore represent an important direction for promoting mental health and facilitating early psychological intervention.

One psychological tendency that may serve as such a preventive psychological resource is adaptive insensitivity. Although the term “insensitivity” may intuitively evoke negative impressions, such as emotional detachment, indifference, or reduced empathy, adaptive insensitivity does not reflect emotional unresponsiveness or avoidance. Rather, it represents a tendency to reduce excessive emotional involvement in response to negative emotional stimuli while preserving adaptive functioning. We define adaptive insensitivity as a relatively stable psychological tendency characterized by attenuated emotional reactivity to excessive negative stimuli while maintaining appropriate emotional awareness, interpersonal functioning, and behavioral flexibility. For conciseness, ‘insensitivity’ is used throughout this study as a shortened reference to adaptive insensitivity.

This conceptualization is consistent with theoretical perspectives suggesting that psychological adaptation involves the regulation of responses to stressful experiences rather than uniformly heightened sensitivity to all environmental information. According to the stress and coping theory, individuals’ responses to stressful events are shaped not only by external stressors but also by their cognitive appraisal processes (Lazarus & Folkman, 1984). Excessive attention to or involvement in negative experiences may therefore amplify psychological burden. Furthermore, the conservation of resources theory proposes that individuals strive to obtain, retain, and protect valuable resources, and stress may occur when these resources are threatened or depleted (Hobfoll, 1989). From this perspective, insensitivity may function as a resource-protective capacity by reducing unnecessary psychological involvement in nonessential stressors while preserving resources for adaptive functioning.

Furthermore, although the term has gained popularity through literary and public discourse, existing discussions have largely remained descriptive and anecdotal, portraying insensitivity as a general philosophy for coping with adversity rather than as a psychologically defined construct. Consequently, its psychological characteristics, underlying mechanisms, and conceptual boundaries remain insufficiently clarified, limiting both theoretical development and empirical investigation. Clarifying the psychological meaning of insensitivity and establishing its conceptual boundaries are therefore essential prerequisites for developing valid measurement instruments and advancing systematic empirical research.

Conceptually, insensitivity is distinguishable from established protective constructs, particularly emotion regulation and psychological resilience. Emotion regulation primarily involves monitoring and modifying emotional responses after emotional reactions have already been activated (Eisenberg & Fabes, 1992; Gross, 1998; Huang & Guo, 2000), whereas insensitivity is proposed to operate at an earlier stage of emotional processing by attenuating the perceived impact of excessive negative stimulation before intense emotional reactions develop. Likewise, although psychological resilience emphasizes successful adaptation and recovery following adversity (Xi et al., 2008, 2012), insensitivity primarily concerns reducing the initial psychological impact of stressors, thereby preventing excessive emotional activation before recovery becomes necessary.

Insensitivity is also conceptually distinct from other related constructs. Mindfulness is generally characterized by intentional attention to present-moment experience with an attitude of openness and nonjudgmental awareness (Bishop et al., 2004; Kabat-Zinn, 2003). It primarily concerns how individuals attend to and relate to their ongoing experiences, whereas insensitivity concerns the degree to which excessive negative stimulation affects subsequent emotional and psychological functioning. Psychological flexibility emphasizes whether individuals can adaptively adjust their responses according to situational demands (Bond et al., 2011; Hayes et al., 2006), whereas insensitivity emphasizes whether individuals can attenuate the excessive psychological impact of negative stimulation when sustained psychological involvement is not necessary. Emotional stability refers to the tendency to maintain relatively consistent and balanced emotional states, with fewer fluctuations in emotional responses across situations (Kuppens et al., 2007). Low neuroticism reflects a broad personality characteristic within the Big Five framework and is associated with lower negative emotionality and emotional reactivity (DeYoung et al., 2007; Grogans et al., 2024). In contrast, insensitivity represents a more specific psychological tendency that is particularly relevant to coping with excessive negative stimulation and does not necessarily imply generally low emotional reactivity across situations. Taken together, these distinctions suggest that insensitivity occupies a conceptually different position from these established constructs.

Rather than replacing these established constructs, insensitivity may complement them by highlighting an earlier and relatively underexplored psychological process involved in maintaining mental health. This perspective extends current understandings of psychological protection beyond emotional recovery and regulation toward the prevention of excessive emotional activation during the initial stage of stress processing. Its unique contribution therefore lies not in eliminating negative emotional responses or promoting generalized emotional detachment, but in emphasizing the potential adaptive value of not becoming excessively affected by negative experiences when such psychological involvement is unnecessary. Furthermore, the three dimensions of insensitivity identified from the qualitative data can be organized and interpreted within the ABC model, which conceptualizes responses to external events as coordinated affective, cognitive, and behavioral processes (Rosenberg, 1960; Zhang & Wang, 2007). From this perspective, insensitivity may be understood as a multidimensional psychological tendency encompassing emotional cognitive management, interpersonal boundary management, and behavioral response management.

From a practical perspective, insensitivity may be particularly valuable in contemporary society, where individuals are increasingly confronted with information overload, frequent social evaluation, and persistent psychological stress. By reducing excessive emotional engagement with negative experiences, this adaptive tendency may help conserve psychological resources, minimize unnecessary emotional exhaustion, and maintain psychological well-being before substantial distress develops. As increasing attention is being devoted to preventive approaches that promote mental health rather than merely alleviate psychological disorders, insensitivity may represent a promising psychological resource for enhancing resilience to everyday stressors and supporting sustainable mental well-being.

Beyond attenuating immediate emotional reactions to negative events, insensitivity may also influence the subsequent cognitive processing of stressful experiences. One potential mechanism underlying this association is rumination, a repetitive pattern of negative thinking that has been consistently identified as a central cognitive vulnerability factor for depression. Extensive evidence indicates that rumination mediates the relationships between stress, maladaptive cognition, and psychological distress (Morrison & O’Connor, 2005), represents one of the strongest predictors of depressive symptoms (Watkins, 2008), and is associated with a wide range of adverse psychological outcomes, including anxiety and emotional maladjustment (Bai et al., 2020; Ma & An, 2019). According to Response Styles Theory (Nolen-Hoeksema et al., 2008), rumination develops when individuals repeatedly attend to and cognitively elaborate on negative experiences and their consequences. Individuals who are highly sensitive to external evaluation, setbacks, or stressful events are more likely to interpret these experiences as threatening and engage in persistent negative thinking. By contrast, individuals with higher levels of insensitivity may perceive excessive negative stimulation as less psychologically threatening, thereby reducing repetitive cognitive elaboration and interrupting the cognitive pathway through which stressful experiences evolve into persistent depressive symptoms. Consequently, insensitivity may contribute to mental health not only by attenuating immediate emotional reactions but also by reducing maladaptive cognitive processing following adverse experiences.

Despite its potential relevance for mental health promotion, insensitivity has not yet been systematically conceptualized within psychological research, and no psychometrically validated instrument is currently available for its assessment. The absence of a theoretically grounded measurement framework has limited empirical investigations into its psychological characteristics, mental health functions, and underlying mechanisms. University students constitute a particularly important population in this regard because they are confronted with multiple developmental challenges, including academic competition, career uncertainty, interpersonal adjustment, and identity development, all of which increase their vulnerability to rumination and depressive symptoms. Developing a reliable and valid measure of insensitivity is therefore an essential prerequisite for systematically investigating its psychological characteristics and its potential role in promoting mental health among university students. Accordingly, the present study aimed to develop and validate the Insensitivity Scale (IS) among Chinese university students. Furthermore, based on Response Styles Theory, this study examined the relationships among insensitivity, rumination, and depressive symptoms and tested whether rumination mediates the association between insensitivity and depression. The findings are expected to contribute to the conceptual clarification and empirical investigation of insensitivity while advancing understanding of its potential role as a preventive psychological resource for mental health.

## 2. Methods

### 2.1. Participants

Word Association and Open-Ended Survey. A convenience sampling method was adopted. Questionnaires were distributed via Wenjuanxing, an online survey platform, to university students from multiple universities across China. A total of 36 participants completed the word association task, and 30 participants completed the open-ended survey.

Semi-Structured Interviews. Purposive sampling was employed to recruit participants, with emphasis placed on selecting information-rich cases that could provide diverse and relevant perspectives (Wu & Huang, 2012). An interview guide was developed in advance based on the research objectives. Prior to the interviews, informed consent was obtained from all participants, who were informed that the interviews would be audio-recorded and transcribed. Each participant took part in an individual face-to-face interview lasting approximately 20 min. Data collection continued until thematic saturation was reached, that is, when newly collected information largely overlapped with existing categories. In total, 18 interview transcripts were obtained, exceeding the minimum recommended sample size of 12 participants for qualitative interview studies. The interviewees included both male and female undergraduate students from the first through fourth academic years.

Scale Development. Convenience sampling was used to recruit participants through Credamo and Naodao, two online platforms supporting psychological survey distribution (G. Chen et al., 2023). Questionnaires were administered to university students aged 18–22 years from multiple universities across 29 provincial-level administrative regions in China, including Anhui, Beijing, Fujian, Gansu, Guangdong, and Guangxi. After data collection, questionnaires were excluded if they had an excessively short completion time, failed attention-check items, inconsistent responses to reverse-scored items, or contained inconsistent responses to repeated items. The remaining questionnaires were retained for analysis. Sample A was used for item analysis and exploratory factor analysis (EFA). Of the 798 questionnaires collected, 705 were valid (effective response rate = 88.35%), with a mean age of 20.98 ± 1.04 years. Sample B was used for confirmatory factor analysis (CFA) and reliability and validity testing. Of the 532 questionnaires collected, 500 were valid (effective response rate = 93.98%), with a mean age of 20.84 ± 1.17 years. Sample C was used to assess test–retest reliability. Of the 150 questionnaires collected, 135 were valid (effective response rate = 90.00%), with a mean age of 20.73 ± 1.22 years.

Mediation Effect Analysis. Using a convenience sampling method, a total of 659 questionnaires were distributed to undergraduate students from multiple universities across 30 provincial-level administrative regions in China, including Zhejiang, Yunnan, and the Xinjiang Uygur Autonomous Region, via the Credamo online survey platform. After data screening based on predefined quality-control criteria (e.g., failure to pass the attention-check item), 600 valid questionnaires were retained, resulting in a valid response rate of 91.05%. Among the participants, 301 were male and 299 were female; 33 were freshmen, 151 sophomores, 237 juniors, and 179 seniors. The mean age was 20.89 ± 1.04 years.

The study was approved by the Institutional Review Board of the first author’s institution (Approval No. XL25082501). All participants provided written informed consent prior to participation.

### 2.2. Questionnaire Development

To clarify the conceptual meaning and content of insensitivity, we first collected words that university students spontaneously associated with the term. Participants were instructed to provide at least 11 descriptive words that came to mind when they heard the term “insensitivity.” Each participant provided 11–12 association words in the task. A total of 422 words were generated. After preliminary screening, clearly meaningless or irrelevant words were removed, and semantically overlapping words were merged, resulting in 186 relatively distinct words. Among these, 35 words had a weighted percentage greater than 0.50%, and 16 had a weighted percentage greater than 1.00%. The three most frequently associated words were “slowness,” “stability,” and “calmness.” Semantically, these words all reflect a tendency to modulate one’s responses to emotional or external stimuli, suggesting that, within university students’ lay understanding, insensitivity is associated with reduced reactivity and psychological stability.

To further explore university students’ understanding and lived experiences of insensitivity, we developed a six-question open-ended survey addressing participants’ understanding, emotional experiences, behavioral manifestations, and perceived effects of insensitivity. The questions were as follows: (1) How do you understand the concept of “insensitivity”? (2) When you respond with insensitivity to a person or situation, what emotional experiences do you have? A frequency analysis based on exact word matching was first conducted across responses. The three most frequently occurring words were “emotion,” “life,” and “things,” suggesting that participants primarily associated insensitivity with emotional experiences and its manifestations in everyday life. Unlike the word association task, which elicited relatively isolated descriptors, the open-ended survey provided contextualized accounts of how participants understood and experienced insensitivity.

Participants generally described insensitivity as a tendency to regulate and adapt emotional responses to everyday situations, including maintaining emotional stability, remaining calm in the face of difficulties, flexibly adapting to changing circumstances, and reducing sensitivity to negative social information. For example, representative responses included: “I think insensitivity is a kind of wisdom in seeming foolishness. One’s emotions are relatively restrained, and one is not easily overwhelmed by intense emotions. One remains relatively stable physically and psychologically and stays calm and composed regardless of the situation”; “People with strong insensitivity are not easily discouraged, have relatively stable emotions, and can block out negative emotions from the outside world without becoming overly sensitive, allowing them to adapt better to various situations in life”; and “I feel that insensitivity means being less sensitive to things happening around you, not constantly worrying about every little thing, and not noticing every instance of hostility. It is a form of psychological self-protection.” These frequently occurring descriptors were subsequently examined in context rather than interpreted solely on the basis of frequency. Their semantic patterns suggested that participants tended to associate insensitivity with reduced reactivity, emotional stability, and a calmer response to external events.

Next, web crawling techniques were employed to collect textual data from social media platforms frequently used by university students. In May 2025, publicly accessible texts related to “insensitivity” were retrieved from Weibo and Baidu, two major Chinese online information-sharing platforms. On Weibo, we searched publicly accessible pages containing popular topics and keywords related to “insensitivity” and collected relevant user-generated posts from the first 50 pages; comments and user-identifying information were not collected. On Baidu, we collected publicly accessible titles and brief descriptions from the first 40 pages of search results using “insensitivity” (钝感力) as the search term. More than 900 text records were obtained across the two platforms. All data were publicly accessible and were collected solely for research purposes without collecting personally identifying or interaction-related information.

The textual data were cleaned and preprocessed by removing irrelevant symbols, stop words, and redundant information, followed by word segmentation and frequency-based analysis. Sentiment analysis was also conducted to examine the emotional valence of naturally occurring discourse surrounding insensitivity. The three most frequent words on Weibo were “myself,” “chaojue” (a popular Chinese internet expression denoting strong admiration or excellence), and “neihao” (psychological self-consumption or excessive internal distress), whereas those on Baidu were “anxiety,” “calmness,” and “difficulties.” These linguistic patterns indicated that discussions of insensitivity were commonly situated in everyday experiences involving the self, psychological distress, emotional states, and difficulties. Sentiment analysis further indicated that online discourse surrounding insensitivity was predominantly positive on both platforms. On Weibo, 81.30% of the texts were classified as positive, 13.20% as neutral, and 5.50% as negative. Similarly, on Baidu, 86.70% were classified as positive, 8.30% as neutral, and 5.00% as negative. Thus, positive sentiment accounted for more than 80% of the texts on both platforms, whereas negative sentiment was relatively uncommon. Taken together, these findings suggest that insensitivity was generally discussed in a favorable rather than a purely negative sense, particularly in relation to maintaining calmness, reducing psychological distress, and coping with everyday difficulties.

Finally, semi-structured in-depth interviews were conducted to obtain more detailed accounts of university students’ understanding and experiences of insensitivity. The interviews were conducted in quiet, interruption-free settings, with the time and location arranged to ensure participants’ safety and convenience. The interview protocol covered four areas: participants’ basic information, their personal understanding of insensitivity, their observations of insensitivity in others and society, and broader reflections on the construct. Representative questions included: “Have you heard of the term ‘insensitivity’?”; “When and in what context did you first encounter the term?”; “How do you understand insensitivity?”; and “When observing others, what cognitive, emotional, or behavioral characteristics would lead you to consider them as having a high level of insensitivity?” After the interviews were completed, the interview materials were organized and reviewed, with particular attention to frequently recurring and conceptually relevant expressions. The first author initially organized and analyzed the interview transcripts and conducted a three-stage coding procedure, consisting of open coding, axial coding, and selective coding. During open coding, the transcripts were examined line by line and conceptually meaningful segments were identified and labeled. This process resulted in 41 initial concepts (free nodes). Although some initial codes were supported by only a single reference point within the interview data, these codes were retained when corresponding expressions or related content were also observed in the open-ended survey, indicating that they were not isolated to a single participant’s account. During axial coding, conceptually related initial codes were compared, organized, and grouped according to their underlying relationships, resulting in six categories. Selective coding was conducted to further integrate and refine the relationships among these categories, resulting in three higher-order categories. The preliminary coding scheme was subsequently discussed with other researchers, and discrepancies were resolved through discussion to reach consensus on the final coding results. Details are presented in Table 1.

The four qualitative sources served complementary purposes. The word association task, open-ended survey, and web-based text analysis were used primarily to identify the semantic content and common lay representations of insensitivity, whereas the semi-structured interviews provided the primary basis for systematically identifying its underlying psychological dimensions through coding. Findings across these sources were compared and integrated during conceptualization and item generation.

Based on the findings from the word association task, open-ended survey, web-based text analysis, and semi-structured interviews, three dimensions of insensitivity were inductively identified from the qualitative data: Emotion Cognitive Management, Interpersonal Boundary Management, and Behavioral Response Management. The ABC model was subsequently used as a theoretical organizing framework to integrate and interpret these empirically derived dimensions. Emotion Cognitive Management refers to an individual’s tendency to process and appraise stressful or unexpected events without excessively amplifying their negative emotional significance, thereby maintaining emotional awareness and psychological balance. This dimension is characterized by neither reacting too quickly nor assigning excessive psychological weight to negative stimuli. Interpersonal Boundary Management refers to the ability to perceive others’ emotions and evaluations while maintaining an appropriate psychological distance, thereby avoiding excessive emotional involvement in others’ experiences or interpersonal conflicts and allocating psychological resources selectively. This dimension is characterized by maintaining appropriate interpersonal distance without disregarding others’ legitimate emotions or needs, and by avoiding the magnification of interpersonal experiences. Behavioral Response Management refers to the ability to remain focused on personally important goals and flexibly adjust behavioral strategies without allowing minor setbacks, negative feedback, or external evaluations to disproportionately disrupt goal-directed behavior. This dimension reflects a behavioral pattern characterized by reduced behavioral interference from low-importance negative stimuli rather than a general capacity for self-regulation or coping.

To translate the qualitative findings into measurable items, conceptually representative and recurrent codes within each category were reviewed to identify the core psychological processes and behavioral manifestations reflected in participants’ accounts. The core meanings reflected by these codes were then translated into concise first-person statements while preserving the meanings expressed by participants. The resulting items were assigned to the dimension that best represented their primary psychological content. Based on this conceptual framework, an initial pool of scale items was generated. One faculty member specializing in psychometrics and two doctoral students in psychology reviewed the items to identify redundancy, inappropriate dimensional classification, and ambiguous wording. Following revisions, an initial version of the scale containing 42 items was obtained. To examine content validity, four psychology faculty members with experience in scale development or validation, two doctoral students in psychology, and two master’s students in psychology were invited to evaluate the relevance of each item to insensitivity using a 4-point scale (1 = not relevant, 4 = highly relevant). The first-round evaluation indicated that most items received ratings of 3 or 4; however, six items each received ratings of ≤2 from two experts, and eleven items each received ratings of ≤2 from one expert. After these 17 items were revised, all experts were invited to re-evaluate the complete item pool. In the second-round evaluation, all items received ratings of 3 or 4, meeting the recommended criteria for content validity. Finally, two master’s students in psychology and twelve undergraduate psychology students were invited to assess the readability of the 42 items by evaluating their clarity, potential ambiguity, and typographical accuracy using a 5-point scale (1 = very poor, 5 = very good). All 14 participants rated every item as either 4 or 5, indicating that the items were readily understood by university students and were clearly and appropriately worded.

### 2.3. Measures

#### 2.3.1. Criterion Measures

In the absence of existing studies that directly identify criterion measures for sensitivity, the present study selected several external criterion measures based on theoretical inferences derived from the construct’s conceptual connotation and functional orientation. The corresponding scales and their usage instructions are presented in Table 2.

#### 2.3.2. Research Instruments

##### Insensitivity Scale (IS)

The Insensitivity Scale (IS), developed by the authors, consists of 15 items across three dimensions. Responses are rated on a five-point Likert scale ranging from 1 (strongly disagree) to 5 (strongly agree), with higher scores indicating higher levels of insensitivity. In the present study, the overall scale demonstrated excellent internal consistency (Cronbach’s α = 0.93). The Cronbach’s α coefficients for the three subscales were 0.87, 0.85, and 0.85, respectively.

##### Center for Epidemiologic Studies Depression Scale (CES-D)

Depressive symptoms were assessed using the Center for Epidemiologic Studies Depression Scale (CES-D) (Ren et al., 2019), which comprises 20 items across four dimensions. Each item is rated on a four-point scale ranging from 0 (rarely or none of the time; less than 1 day) to 3 (most or all of the time; 5–7 days). Higher scores indicate more severe depressive symptoms. In the present study, the Cronbach’s α coefficient for the scale was 0.92.

##### Ruminative Responses Scale (RRS)

Rumination was measured using the Ruminative Responses Scale (RRS), as adapted by Han and Yang (2009) from the original measure developed by Nolen-Hoeksema. The scale contains 15 items across three dimensions. Responses are rated on a four-point Likert scale ranging from 1 (never) to 4 (often), with higher scores indicating greater levels of rumination. The Cronbach’s α coefficient was 0.93 in the present study.

##### College Student’s Perceived Social Mindfulness Scale (C-PSMS)

Perceived social mindfulness was assessed using the College Student’s Perceived Social Mindfulness Scale (C-PSMS) (Lei et al., 2024), which consists of 18 items. Participants respond on a five-point Likert scale ranging from 1 (strongly disagree) to 5 (strongly agree). Higher scores indicate higher levels of perceived social mindfulness. In the present study, the scale demonstrated excellent internal consistency, with a Cronbach’s α coefficient of 0.94.

### 2.4. Statistical Analysis

NVivo was used to code the interview transcripts and establish node indices for qualitative analysis. SPSS was employed to conduct item analysis and exploratory factor analysis (EFA) of the preliminary version of the scale. AMOS was used to perform confirmatory factor analysis (CFA) and evaluate the reliability and validity of the final version of the scale.

Data were analyzed using IBM SPSS Statistics, Version 27.0 (IBM Corp., Armonk, NY, USA). Pearson correlation analyses were conducted to examine the associations among the study variables. All variables were standardized prior to analysis. The mediation effect of rumination on the relationship between insensitivity and depressive symptoms was examined using the PROCESS macro for SPSS. Common method bias was assessed using Harman’s single-factor test. An unrotated exploratory factor analysis extracted 10 factors with eigenvalues greater than 1. The first factor accounted for 36.0% of the total variance, which was below the commonly accepted threshold of 40%, suggesting that no single factor accounted for the majority of the variance.

## 3. Results

### 3.1. Item Analysis

Item analysis was conducted using both the extreme-group method and the item–total correlation method. First, the extreme-group method was employed. Participants were ranked according to their total scores on the scale, and the highest 27% and lowest 27% were classified as the high-score and low-score groups, respectively. Independent-samples *t*-tests were then conducted to compare the two groups on each item. The results showed that all 42 items significantly differentiated between the high-score and low-score groups (*p* < 0.01), indicating satisfactory item discrimination. Second, item–total correlation analyses were performed. The corrected item–total correlation coefficients ranged from 0.55 to 0.77, and all correlations were statistically significant (*p* < 0.01), suggesting good homogeneity between each item and the overall scale. Taken together, all items demonstrated satisfactory discriminatory power and were therefore retained for subsequent analyses.

### 3.2. Exploratory Factor Analysis

The suitability of the data for exploratory factor analysis (EFA) was first evaluated. The results indicated excellent sampling adequacy (Kaiser–Meyer–Olkin, KMO = 0.98), and Bartlett’s test of sphericity was significant, χ^2^ = 15,618.42 (*p* < 0.001), suggesting that the data were appropriate for factor analysis. Exploratory factor analysis was then conducted using principal axis factoring with direct oblimin rotation. The initial analysis extracted four factors with eigenvalues greater than 1, accounting for 46.64% of the total variance. Items were subsequently removed based on the following criteria: communalities below 0.40, factor loadings below 0.40, cross-loading differences smaller than 0.20, or fewer than three items loading on a factor. Item retention and deletion were also guided by theoretical considerations, including the conceptual relevance and redundancy of item content, to ensure that item reduction did not compromise the conceptual coverage of the construct. After several rounds of item refinement, a three-factor solution comprising 15 items was retained. The final model explained 53.00% of the total variance. The retained items covered all three dimensions identified during the qualitative development process, Emotion Cognitive Management, Interpersonal Boundary Management, and Behavioral Response Management, with five items representing each dimension. Detailed factor loadings are presented in Table 3.

Specifically, the initial pool of 42 items underwent an iterative item reduction process based on a combination of statistical and conceptual considerations. In the initial screening stage, Q11, Q18, Q20, Q21, Q27, and Q29 were removed because of inadequate communalities and/or factor loadings. In subsequent exploratory analyses, Q35 and Q37 were removed because of substantial cross-loadings and ambiguous factor attribution, while Q19, Q30, Q38, Q39, Q40, Q41, and Q42 were removed because they did not contribute sufficiently to a stable and interpretable factor structure. Q1, Q5, Q8, Q9, Q10, Q12, and Q13 were subsequently excluded because of unclear factor attribution. In the final refinement stage, Q22, Q24, Q25, Q26, and Q28 were removed because their loading patterns were relatively weak or less clearly aligned with the emerging factors, thereby improving the parsimony and interpretability of the three-factor solution. Following these iterative procedures, 27 items were removed and 15 items were retained for the final scale.

### 3.3. Confirmatory Factor Analysis

**Figure 1:**
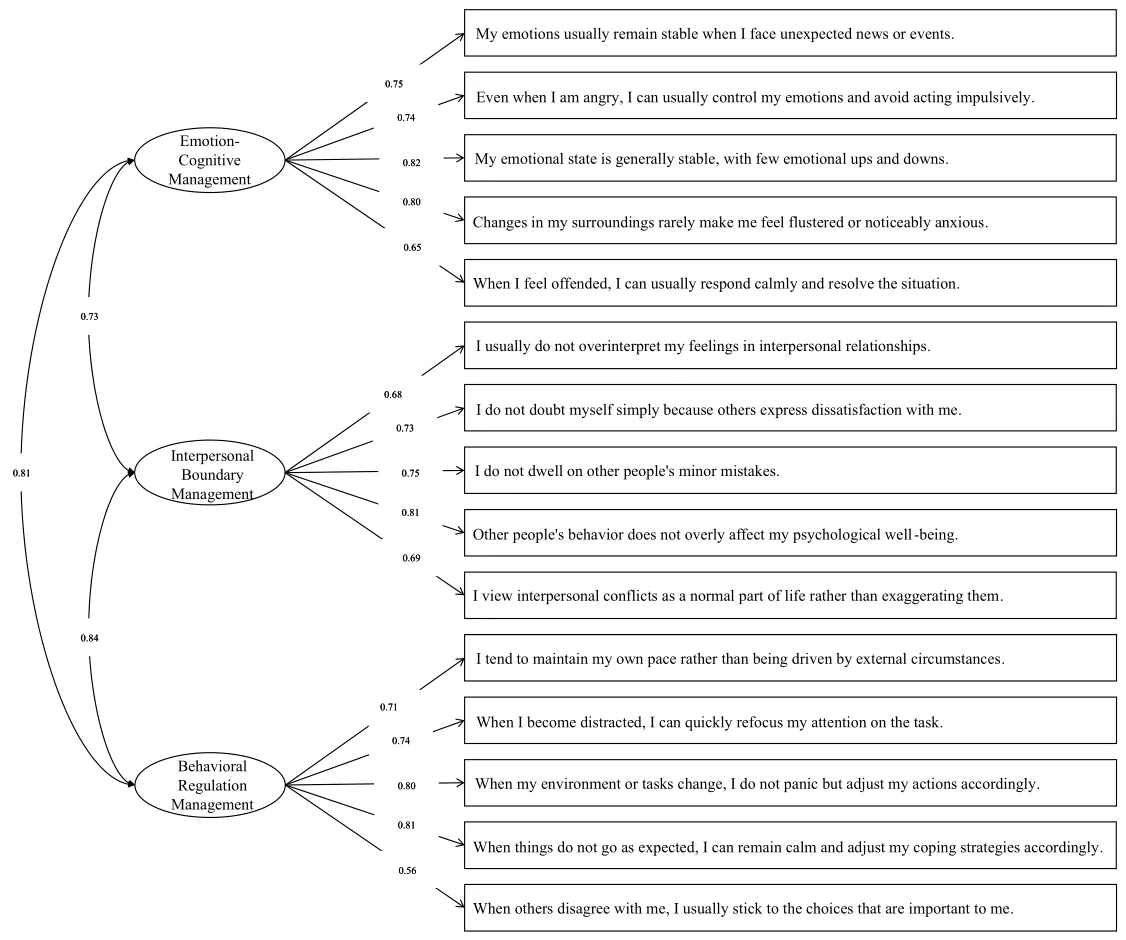
Confirmatory factor analysis model of the Insensitivity Scale.

A confirmatory factor analysis (CFA) was conducted to validate the three-factor structure identified through the exploratory factor analysis. The three factors were specified as latent variables, with the corresponding 15 items serving as observed indicators. The results indicated a good model fit to the data (χ^2^/df = 2.15, NFI = 0.95, IFI = 0.98, TLI = 0.97, CFI = 0.98, RMSEA = 0.05), supporting the proposed three-factor structure. The standardized model is presented in Figure 1.

### 3.4. Validity Assessment

#### 3.4.1. Factorial Validity and Alternative Model Comparisons

Confirmatory factor analysis (CFA) was conducted to further examine the factorial structure of the 15-item Insensitivity Scale. Three alternative models were tested: a one-factor model, a correlated three-factor model, and a second-order three-factor model. The one-factor model showed relatively poor fit to the data (χ^2^/df = 6.09, CFI = 0.88, TLI = 0.86, RMSEA = 0.10). In contrast, both the correlated three-factor model and the second-order three-factor model demonstrated good fit (χ^2^/df = 2.15, CFI = 0.98, TLI = 0.97, RMSEA = 0.05).

Although the correlated three-factor and the second-order three-factor models showed identical fit indices, they represent different theoretical structures. In the correlated three-factor model, the correlations among the three first-order factors ranged from 0.73 to 0.84, indicating substantial associations among the three dimensions. In the second-order model, the standardized loadings of the higher-order Insensitivity factor on the three first-order factors were 0.84, 0.87, and 0.96, respectively. These findings support the conceptualization of insensitivity as a higher-order construct comprising three related dimensions: Emotion Cognitive Management, Interpersonal Boundary Management, and Behavioral Response Management.

Given the good fit of the second-order model, the substantial higher-order factor loadings, and the theoretical conceptualization of insensitivity as a multidimensional construct with a common underlying factor, the total score was considered an indicator of overall insensitivity, while the three subscale scores were retained to represent the specific dimensions of the construct.

#### 3.4.2. Confirmatory Factor Analysis and Construct Distinctiveness

To examine whether insensitivity was empirically distinguishable from conceptually related constructs, we compared a one-factor model, in which all items measuring insensitivity, resilience, regulatory emotional self-efficacy, and emotional vulnerability loaded onto a single latent factor, with a four-factor model, in which the four constructs were specified as distinct but correlated latent factors. The one-factor model showed relatively poor fit to the data (χ^2^/df = 3.95, CFI = 0.74, TLI = 0.73, RMSEA = 0.08), whereas the four-factor model demonstrated a comparatively better fit (χ^2^/df = 3.19, CFI = 0.81, TLI = 0.80, RMSEA = 0.07). The substantially improved fit of the four-factor model over the one-factor model provides preliminary evidence that insensitivity is not adequately represented as a single construct with resilience, regulatory emotional self-efficacy, and emotional vulnerability.

Discriminant validity was further assessed using the heterotrait–monotrait (HTMT) ratio of correlations. As shown in Table 4, the HTMT values ranged from 0.80 to 0.89. All values were below the 0.90 criterion, although two values exceeded the more conservative 0.85 threshold, indicating acceptable discriminant validity with some degree of conceptual overlap among the constructs.

#### 3.4.3. Convergent and Criterion-Related Validity

Criterion-related validity was examined by assessing the correlations between the Insensitivity Scale and five criterion variables. As shown in Table 5, insensitivity was significantly correlated with all five criterion variables, with the directions of the associations generally consistent with the hypothesized relationships. These findings support the criterion-related validity of the Insensitivity Scale. The correlations with resilience, regulatory emotional self-efficacy, and emotional vulnerability were relatively high and should therefore be interpreted with some caution. Convergent validity was further evaluated using standardized factor loadings, the average variance extracted (AVE), and composite reliability (CR). The standardized factor loadings ranged from 0.56 to 0.82, all exceeding the recommended threshold of 0.50. The AVE values for the overall scale and the three subscales were 0.55, 0.57, 0.54, and 0.53, respectively, all above the recommended criterion of 0.50. The corresponding CR values were 0.95, 0.87, 0.85, and 0.85, respectively, all exceeding the recommended threshold of 0.70.

#### 3.4.4. Incremental Validity

Incremental validity was examined using a series of hierarchical regression analyses to determine whether the Insensitivity Scale explained additional variance in depressive symptoms beyond conceptually related constructs. First, resilience was entered as the predictor in Step 1, followed by insensitivity in Step 2. Resilience explained 22.0% of the variance in depressive symptoms (R^2^ = 0.22), and adding insensitivity significantly increased the explained variance by 3.7% (ΔR^2^ = 0.037, ΔF(1, 497) = 24.515, *p* < 0.001), yielding a final R^2^ of 0.256. A similar analysis was conducted with regulatory emotional self-efficacy. Regulatory emotional self-efficacy explained 21.8% of the variance in depressive symptoms (R^2^ = 0.218), and the addition of insensitivity accounted for a further 3.3% of the variance (ΔR^2^ = 0.033, ΔF(1, 497) = 22.205, *p* < 0.001), with the final model explaining 25.1% of the variance (R^2^ = 0.251). When emotional vulnerability was entered first, it explained 34.9% of the variance in depressive symptoms (R^2^ = 0.349). Adding insensitivity increased R^2^ to 0.353, but this change was not statistically significant (ΔR^2^ = 0.004, ΔF(1, 497) = 3.383, *p* = 0.066).

Finally, to examine the incremental contribution of insensitivity when all three related constructs were considered simultaneously, resilience, regulatory emotional self-efficacy, and emotional vulnerability were entered in Step 1, followed by insensitivity in Step 2. The three constructs jointly explained 36.2% of the variance in depressive symptoms (R^2^ = 0.362, adjusted R^2^ = 0.358). Adding insensitivity did not further improve the model (ΔR^2^ < 0.001, ΔF(1, 495) = 0.122, *p* = 0.727). In the final model, insensitivity was not a significant predictor of depressive symptoms (β = 0.025, *p* = 0.727). Taken together, the findings provide mixed evidence regarding the incremental validity of insensitivity. Insensitivity explained additional variance in depressive symptoms beyond resilience and regulatory emotional self-efficacy when each construct was examined separately. However, its incremental contribution was small and non-significant after emotional vulnerability was controlled, and it did not explain additional variance when all three related constructs were considered simultaneously. These findings suggest that the incremental validity of insensitivity is limited in the present sample and that substantial overlap with closely related constructs, particularly emotional vulnerability, should be acknowledged.

### 3.5. Reliability Analysis

The Insensitivity Scale demonstrated good reliability. The overall Cronbach’s α coefficient was 0.93, while the Cronbach’s α coefficients for the three subscales, Emotion Cognitive Management, Interpersonal Boundary Management, and Behavioral Response Management, were 0.87, 0.85, and 0.85, respectively. Furthermore, the test–retest reliability coefficient for the overall scale after one month was 0.87 (*p* < 0.01), indicating good temporal stability.

### 3.6. Correlations Among the Study Variables

The results of the Pearson correlation analyses showed that the mean score for insensitivity was 4.05 ± 0.67, the mean score for depressive symptoms was 0.51 ± 0.42, and the mean score for rumination was 1.89 ± 0.55. Insensitivity was negatively correlated with both depressive symptoms (*r* = −0.71, *p* < 0.01) and rumination (*r* = −0.61, *p* < 0.01). In addition, depressive symptoms were positively correlated with rumination (*r* = 0.78, *p* < 0.01).

### 3.7. Mediating Effect of Rumination

**Figure 2:**
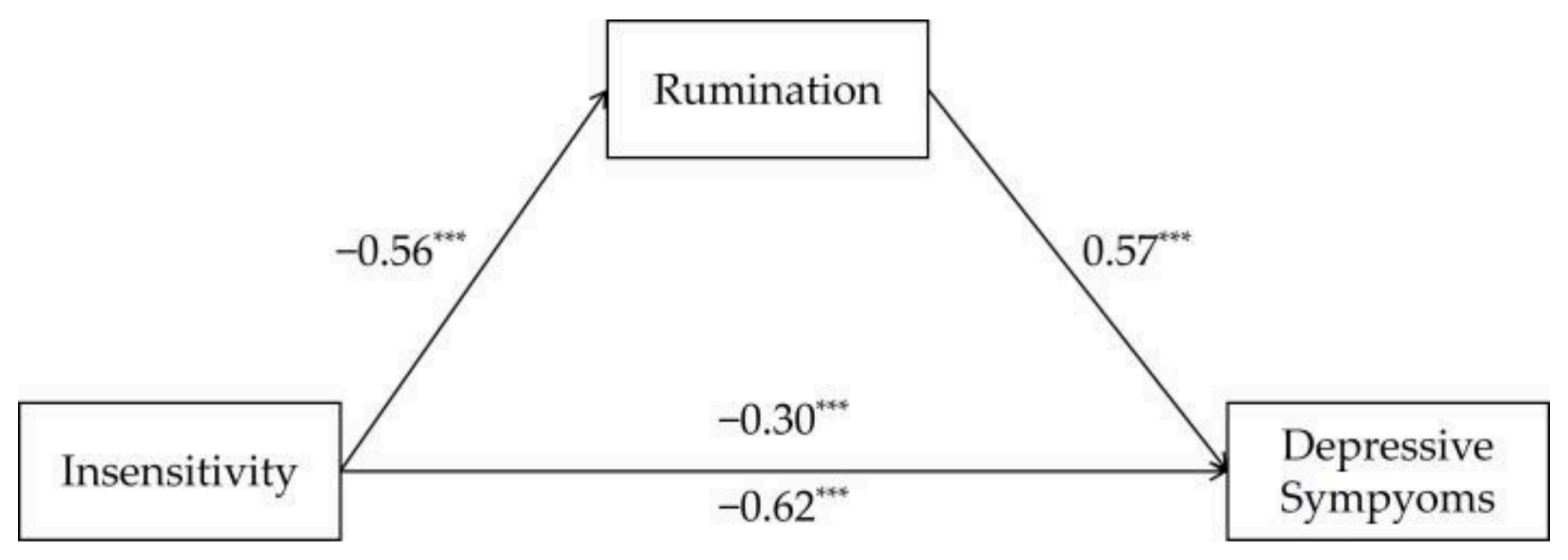
Mediation model of the relationship between insensitivity and depressive symptoms through rumination. (Note. *** *p* < 0.001).

All core variables were standardized before the analyses. The mediating effect of rumination on the association between insensitivity and depressive symptoms was examined using Model 4 of the PROCESS macro developed by Hayes for SPSS. The indirect effect was estimated using the bootstrap method with 5000 resamples. As shown in Figure 2, rumination significantly mediated the relationship between insensitivity and depressive symptoms. Insensitivity was negatively associated with rumination (a = −0.56, SE = 0.03, *t* = −19.05, *p* < 0.001), indicating that higher levels of insensitivity were associated with lower levels of rumination. In turn, rumination was positively associated with depressive symptoms after controlling for insensitivity (b = 0.57, SE = 0.03, *t* = 20.90, *p* < 0.001). The total effect of insensitivity on depressive symptoms was negative and significant (c = −0.62, SE = 0.03, *t* = −24.23, *p* < 0.001). After rumination was included in the model, the direct effect remained significant and negative (c′ = −0.30, SE = 0.02, *t* = −12.29, *p* < 0.001). The indirect effect was calculated as ab = (−0.56) × (0.57) = −0.32, with a BootSE of 0.03, accounting for 52% of the total effect. The 95% confidence interval ranged from −0.38 to −0.26, indicating that the indirect effect was statistically significant because the confidence interval did not include zero.

## 4. Discussion

In the present study, lexical association, open-ended surveys, and web crawling were employed to identify the core concepts that college students associate with insensitivity, as well as the emotional connotations and value orientations attached to this construct in the public discourse. Subsequently, in-depth interviews were conducted to clarify the underlying psychological structure of insensitivity among college students. Guided by the ABC model, the interview data were systematically coded and categorized, resulting in a three-factor structure. The first dimension, Emotion Cognitive Management, reflects an individual’s ability to maintain emotional stability when experiencing stress, failure, or negative evaluation, avoid excessive immersion in negative experiences, and interpret situations in a relatively rational manner. The second dimension, Interpersonal Boundary Management, refers to the ability to maintain appropriate psychological boundaries in interpersonal interactions, avoid excessive concern about others’ opinions, and prevent unnecessary emotional depletion arising from complex interpersonal relationships. The third dimension, Behavioral Response Management, reflects the ability to maintain an appropriate pace of action and remain goal-directed when dealing with everyday tasks, rather than responding impulsively or hastily to changing circumstances. These three dimensions are not independent but jointly constitute the overall functional structure of insensitivity. Together, these dimensions characterize a positive psychological tendency associated with reduced psychological depletion, greater internal stability, and more adaptive functioning in complex environments. This conceptualization is also consistent with the core semantic themes of “stability” and “calmness” identified in the preliminary text analysis.

Subsequently, an initial item pool was generated based on the coding results. After satisfactory content validity and comprehensibility had been established, the formal version of the scale was administered and evaluated following standard psychometric procedures, including item analysis, exploratory factor analysis, confirmatory factor analysis, and reliability and validity testing. The findings indicated that the final scale consisted of 15 items across three dimensions. These results suggest that the Insensitivity Scale possesses a stable factor structure and satisfactory measurement reliability, providing a reliable instrument for assessing insensitivity among university students. Moreover, both the exploratory and confirmatory factor analyses consistently supported the proposed three-factor structure, indicating that Emotion Cognitive Management, Interpersonal Boundary Management, and Behavioral Response Management are conceptually distinct yet collectively represent the higher-order construct of insensitivity. These findings are also consistent with the preliminary qualitative analyses, suggesting that insensitivity should not be understood simply as reduced sensitivity. Rather, it represents a multidimensional psychological disposition encompassing emotional and cognitive regulation, interpersonal functioning, and behavioral regulation.

The quantitative findings further suggest that insensitivity is closely related to established constructs while potentially capturing some distinguishable aspects of adaptive psychological functioning. When examined separately, insensitivity explained additional variance in depressive symptoms beyond resilience and regulatory emotional self-efficacy. However, its incremental contribution was no longer significant when emotional vulnerability was controlled, and it did not provide additional explanatory value when all three related constructs were considered simultaneously. These findings indicate that the incremental validity of IS is limited in the present study and that substantial overlap exists between IS and closely related constructs, particularly emotional vulnerability. Although the incremental validity findings were mixed, the conceptual focus of IS warrants further consideration. Emotional vulnerability primarily concerns an individual’s susceptibility to being adversely affected by negative emotional experiences, whereas IS focuses on the tendency to limit unnecessary psychological engagement with personally relevant negative stimuli while maintaining emotional awareness and adaptive functioning. Thus, the conceptual distinction lies not simply in whether an individual is affected by negative experiences, but in the extent to which psychological involvement with such experiences is selectively limited when it is unnecessary or disproportionate. Nevertheless, the substantial association between IS and emotional vulnerability indicates that this conceptual distinction is not sufficient to establish empirical independence. Further research using stronger tests of discriminant and incremental validity is therefore needed to determine whether IS captures unique aspects of adaptive psychological functioning. The better fit of the multi-factor measurement model than the one-factor model provides some evidence for structural differentiation between IS and related constructs. However, this structural evidence should not be interpreted as demonstrating complete empirical independence, particularly given the limited incremental validity observed in the regression analyses. Taken together, IS may be better understood as a related but conceptually specific pattern of psychological functioning, whose unique contribution remains to be clarified through further research.

Beyond its overlap with established psychological constructs, the conceptual boundaries of insensitivity also warrant consideration, particularly with respect to its interpersonal dimension. It is important to note that Interpersonal Boundary Management should be distinguished from empathy deficits, interpersonal apathy, and indifference to others’ suffering. Maintaining psychological distance does not imply ignoring others’ legitimate needs, distress, or experiences of exclusion and discrimination, nor does it imply tacitly accepting discriminatory or exclusionary treatment; rather, it involves remaining aware of others’ emotions and interpersonal cues while regulating excessive emotional involvement that may lead to unnecessary psychological depletion. In this sense, insensitivity may be understood as a psychological boundary that is potentially associated with emotional stability while preserving basic interpersonal awareness and appropriate responsiveness. However, when psychological distance becomes excessive and is accompanied by substantially lower sensitivity or responsiveness to others’ legitimate needs, suffering, or experiences of discrimination, such excessive distancing may instead resemble forms of “privileged insensitivity.” The items are primarily focused on individuals’ psychological and behavioral responses to personally relevant negative experiences and interpersonal stressors, rather than attitudes toward social injustice, discrimination, or the suffering of others. Accordingly, the psychological distance or “not taking things personally” identified in the qualitative phase refers to limiting unnecessary psychological involvement with personally relevant negative experiences, rather than indifference toward others or social problems.

Future research should therefore examine whether Interpersonal Boundary Management can be empirically distinguished from constructs characterized by interpersonal coldness, callousness, and reduced empathy, particularly Dark Triad traits such as psychopathy. More broadly, the adaptive value of psychological distance may depend on the social context in which interpersonal stress, exclusion, or discrimination occurs. The present study primarily adopts an individual-level psychological perspective and does not explicitly examine the sociological, organizational, or institutional conditions that may give rise to the need for interpersonal boundary management and shape its consequences. Thus, insensitivity should be understood as an individual-level psychological resource rather than as a substitute for addressing harmful interpersonal or institutional conditions.

Importantly, the potential application of the IS should also be approached with caution to avoid unintended stigmatization. A lower IS score should not be interpreted as evidence of maladjustment, psychological weakness, incompetence, or inadequate resilience, nor should the scale be used to screen, rank, or select individuals on the basis of their sensitivity. For individuals with heightened sensory or emotional sensitivity, including those who identify with high sensitivity, as well as individuals with neurodevelopmental conditions or characteristics such as autism spectrum disorder (ASD) or attention-deficit/hyperactivity disorder (ADHD), encouraging them to become “less sensitive” may risk pathologizing or devaluing characteristics that may be relatively stable and not readily amenable to simple modification. Such an approach could inadvertently reinforce ableist assumptions by placing the responsibility for adaptation primarily on the individual. Therefore, the IS should be used proactively and cautiously as one source of information for understanding individual–environment fit and informing appropriate environmental adjustments, rather than as a measure of individual competence, resilience, or overall adaptability. In educational or organizational settings, IS scores, when considered alongside contextual and functional information, may help identify situations in which additional environmental support or reasonable accommodations could be beneficial, such as access to quieter spaces, flexibility in assignment arrangements, or other context-sensitive adjustments. The goal should be to improve the fit between individuals and their environments rather than to require individuals with lower IS to conform to a presumed “optimal” level of insensitivity.

Previous discussions of insensitivity have largely remained at the level of literary narratives, practical experience, or public discourse, with limited efforts devoted to establishing a systematic psychological definition or a standardized measurement instrument. Consequently, existing studies have been constrained by inconsistencies in construct definition and limited comparability across findings. The Insensitivity Scale developed in the present study provides a quantitative assessment of university students’ levels of insensitivity by evaluating their performance across three dimensions: Emotion Cognitive Management, Interpersonal Boundary Management, and Behavioral Response Management. Using a Likert-type rating format, the scale enables the quantification of individual differences in insensitivity. The development of this instrument not only provides a valid measurement tool for future research but also lays a methodological foundation for further empirical investigations of the construct. In addition, it facilitates the assessment of the current level of insensitivity among university students and creates opportunities to further examine its associations with psychological health, stress adaptation, emotion regulation, and social functioning. From a practical perspective, the scale may also serve as a useful assessment tool for educators and mental health professionals. By identifying the specific domains in which university students demonstrate greater psychological stability or, conversely, are more vulnerable to excessive sensitivity, emotional overinvolvement, or behavioral depletion, practitioners may better understand common patterns of psychological adaptation in this population. Such information may help university students gain greater insight into their own responses to stress and emotion regulation processes and may provide a basis for reflecting on more stable, rational, and psychologically well-bounded patterns of functioning.

In addition, the present study found that rumination statistically mediated the association between insensitivity and depressive symptoms. Specifically, insensitivity was negatively associated with depressive symptoms, and the mediation analysis further indicated a significant indirect association between insensitivity and depressive symptoms through rumination. According to Response Styles Theory, individuals who repeatedly focus on negative emotions and continuously dwell on the causes and consequences of stressful or adverse experiences are more likely to become trapped in repetitive negative thinking, thereby exacerbating and maintaining depressive symptoms (Nolen-Hoeksema, 1991; Nolen-Hoeksema et al., 2008). In contrast, the core characteristics of insensitivity, including Emotion Cognitive Management, Interpersonal Boundary Management, and Behavioral Response Management, may be associated with less excessive attention to negative events and less repetitive cognitive processing. Accordingly, higher levels of insensitivity were associated with less persistent rumination, which was in turn associated with lower levels of depressive symptoms. These findings suggest that rumination may represent an important cognitive pathway underlying the association between insensitivity and depressive symptoms. However, given the cross-sectional design of the present study, these findings should be interpreted as statistical associations rather than evidence of a causal process. From a practical perspective, the observed associations between insensitivity, rumination, and depressive symptoms may provide a basis for further investigation of potential psychological factors relevant to university students’ mental health.

Importantly, understanding insensitivity as an individual psychological resource should not be interpreted as relieving universities or broader social institutions of their responsibility to address environmental sources of stress and information overload. In university settings, psychological burden may arise not only from individual differences in coping but also from structural and environmental conditions, such as intensive curricula, competing academic demands, and career-related pressures. Universities therefore retain an important responsibility to identify and reduce modifiable environmental stressors rather than relying solely on students’ individual coping capacities. Accordingly, insensitivity should not be used to shift responsibility for such conditions onto individuals by implying that students simply need to become less sensitive. This consideration is particularly important for students with heightened sensory sensitivity or neurodevelopmental characteristics, for whom appropriate institutional support and reasonable accommodations may be necessary. Insensitivity should therefore be regarded as one potential form of individual self-regulation that may be associated with adaptive psychological functioning, rather than as a substitute for institutional efforts to reduce modifiable environmental stressors or address structural sources of psychological burden.

Despite these contributions, the present study has several limitations. First, the participants were limited to university students, and although they were recruited from multiple universities, their representativeness remains limited. In addition, convenience sampling through online survey platforms such as Credamo and Wenjuanxing may have introduced sampling bias, as individuals who voluntarily participate in online surveys may systematically differ from those who do not participate. Second, all variables were assessed using self-report measures, which may be susceptible to self-report and social desirability biases and may not fully capture participants’ actual behavioral responses. The use of a common measurement method may also have contributed to common method variance. Although Harman’s single-factor test did not indicate a dominant single factor, the limited sensitivity of this procedure to common method variance should be acknowledged. Future research should therefore recruit more diverse and representative samples using more rigorous sampling approaches where feasible and incorporate behavioral indicators and other-rated measures, such as peer reports, to provide a more comprehensive assessment of insensitivity. The applicability and psychometric stability of the Insensitivity Scale should also be examined across different age groups and occupational populations. Third, the present study focused exclusively on Chinese university students, and the conceptualization of adaptive insensitivity may be partly shaped by cultural context. In particular, Interpersonal Boundary Management may reflect interpersonal norms that may be particularly salient in collectivist cultural contexts, where interpersonal harmony and others’ evaluations may receive greater emphasis. Therefore, the meaning and adaptive function of insensitivity may vary across cultures, and cross-cultural validation of the Insensitivity Scale is needed in future research. Finally, longitudinal and experimental research is needed to examine the antecedents, development, and temporal dynamics of insensitivity. Such efforts would contribute to a more comprehensive understanding of the role of insensitivity in psychological adaptation and provide stronger theoretical and empirical foundations for future research and intervention development.

## 5. Conclusions

This study represents one of the first attempts to systematically conceptualize and operationalize insensitivity as a psychological construct. Guided by the ABC model, the present study developed and validated the Insensitivity Scale (IS), providing a psychometrically sound instrument for assessing insensitivity among Chinese university students. The findings support a multidimensional conceptualization of insensitivity comprising Emotion Cognitive Management, Interpersonal Boundary Management, and Behavioral Response Management, and provide a basis for further examining its relationship with established constructs such as emotion regulation and psychological resilience.

Furthermore, the findings demonstrated that insensitivity was negatively associated with depressive symptoms, with rumination statistically mediating this association. These findings suggest that rumination may represent an important cognitive pathway underlying the association between insensitivity and depressive symptoms and provide preliminary evidence for the potential relevance of insensitivity to adaptive psychological functioning and mental health.

Overall, this study contributes to the conceptualization and empirical investigation of insensitivity while providing an instrument with satisfactory psychometric properties for future research. The findings further suggest that insensitivity may represent a potentially adaptive psychological resource that warrants further investigation in relation to mental health, rumination, and depressive symptoms among university students. Future research should continue to examine the developmental mechanisms and longitudinal trajectories of insensitivity, its cross-cultural applicability, and its relationship with closely related psychological constructs to further clarify its role in psychological adaptation across diverse populations.

## Figures and Tables

**Table 1:** Coding Results.

Theme	Category	Included Codes (Frequency)
Emotion Cognitive Management	Emotional Stability 6	Remaining calm in difficult situations (17), Emotional stability (13), Inner calm (3), Composure (2), Facing everything calmly (1), Not easily anxious (1)
	Cognitive Acceptance 8	Peaceful mindset (10), Acceptance (7), Rapid emotional recovery (3), Optimism (2), Open-mindedness (2), Self-comfort (2), Not striving for perfection (1), Not putting excessive pressure on oneself (1)
Interpersonal Boundary Management	Psychological Distance 10	Being insensitive (13), Not caring (8), Not taking things personally (7), Letting things go (4), Not dwelling on issues (4), Thick-skinned (3), Not overthinking (2), Realizing things later (2), Tolerance (2), Keeping relationships simple (1)
	Selective Engagement 3	Not magnifying minor conflicts (1), Not dwelling on trivial details (1), Focusing on what matters most (1)
Behavioral Response Management	Self-Focus 7	Staying focused (11), Not being distracted by external influences (6), Slow to react (4), Facing challenges positively (3), Deliberate pace (3), Adapting to change (2), Being organized and methodical (1)
	Continuous Growth 7	Determination (7), Facing difficulties courageously (5), Strong stress tolerance (3), Resilience (3), Growing through setbacks (2), Persistence (2), Not giving up easily (1)

**Table 2:** Criterion Measures Used for the Validation of the Sensitivity Scale.

Measure	Source	Items	Dimensions	Response Format	Reverse-Scored Items	Cronbach’s α
Brief Resilience Scale	(W. Chen et al., 2020)	6	1	1 (Strongly disagree)–5 (Strongly agree)	Yes (3 items)	α = 0.88
Regulatory Emotional Self-Efficacy Scale	(Tian, 2012)	12	4	1 (Very poor ability)–5 (Very strong ability)	No	α_total_ = 0.80
Affective Vulnerability Scale	(Yan et al., 2025)	16	4	1 (Strongly inconsistent)–4 (Strongly consistent)	No	α_total_ = 0.90
Depression Anxiety Stress Scales-21 (DASS-21)	(Gong et al., 2010; Lu et al., 2020)	21	3	0 (Did not apply to me at all)–3 (Applied to me very much or most of the time)	No	α_depression_ = 0.84 α_anxiety_ = 0.87

**Table 3:** Item Loadings and Communalities (Pattern Matrix).

Item	Factor	Communality
X2	0.66	0.62
X3	0.81	0.62
X4	0.55	0.63
X6	0.66	0.63
X7	0.65	0.51
X14	0.57	0.45
X15	0.59	0.50
X16	0.41	0.42
X17	0.79	0.63
X23	0.46	0.44
X31	0.69	0.55
X32	0.53	0.47
X33	0.66	0.54
X34	0.65	0.53
X36	0.58	0.41

**Table 4:** HTMT Ratios Among the Constructs.

Variable	Resilience	Regulatory Emotional Self-Efficacy	Emotional Vulnerability
Insensitivity	0.85	0.89	0.82
Resilience		0.88	0.80
Regulatory Emotional Self-Efficacy			0.80

**Table 5:** Criterion-Related Validity of the Insensitivity Scale (r).

Variable	Resilience	Regulatory Emotional Self-Efficacy	Emotional Vulnerability	Depressive Symptoms	Anxiety
Insensitivity	0.77 **	0.80 **	−0.74 **	−0.48 **	−0.52 **

Note. ** *p* < 0.05.

## Data Availability

The data that support the findings of this study are available from the corresponding author upon reasonable request.
